# Neurotrophic Activity of the Carrageenophyte* Kappaphycus alvarezii* Cultivated at Different Depths and for Different Growth Periods in Various Areas of Indonesia

**DOI:** 10.1155/2018/1098076

**Published:** 2018-10-18

**Authors:** Gabriel Tirtawijaya, Maria Dyah Nur Meinita, Bintang Marhaeni, Md. Nazmul Haque, Il Soo Moon, Yong-Ki Hong

**Affiliations:** ^1^Department of Biotechnology, Pukyong National University, Nam-gu, Busan 48513, Republic of Korea; ^2^Faculty of Fisheries and Marine Science, Jenderal Soedirman University, Purwokerto 53123, Indonesia; ^3^Department of Anatomy, College of Medicine, Dongguk University, Gyeongju 38066, Republic of Korea

## Abstract

The carrageenophyte* Kappaphycus alvarezii* (Rhodophyta) has neurotrophic activity in primary hippocampal neurons. This seaweed is abundant and easily cultivated in tropical coastal areas. To determine the best growth conditions for neurotrophic activity, thalli were grown at different depths and for different periods in various areas of Indonesia. Neurotrophic activity was measured based on the number of primary neurites, the total length of the primary neurites, and the length of the longest neurite.* K*.* alvarezii* had higher neurotrophic activity than carrageenophytes* K*.* striatum* and* Eucheuma denticulatum *cultured under the same conditions.* K*.* alvarezii* grown at the surface for 45 days had higher (1.4- to 1.8-fold) neurotrophic activity than thalli grown at depth (2 m) or harvested sooner (15 days) (*P* < 0.05). Relatively high activities were detected in thalli cultured at Ternate and Garut, Indonesia. Therefore, from a commercial perspective, the culture conditions at the surface for 45 days were optimal for the production of both neurotrophic compounds and carrageenan.* K*.* alvarezii* produced neurotrophic compounds under various environmental conditions, although some conditions were optimal.

## 1. Introduction

Neurodegenerative disorders are a crucial threat to human health, especially in the elderly. These disorders are characterized by gradual degeneration of the structure and function of the central or peripheral nervous system [1]. The most common neurodegenerative disorder is Alzheimer's disease. People with Alzheimer's disease have memory dysfunction and hippocampal atrophy [2, 3]. Neurotrophic activity can prevent cell death in neurodegenerative processes and play key roles in the survival, differentiation, synaptogenesis, and maturation of affected neurons in Alzheimer's disease [4].

Macroalgae are used in health foods, diet supplements, and other useful compounds. The edible rhodophyte* Kappaphycus alvarezii *produces a hydrocolloid carrageenan that is used as a food additive and a gelling, emulsifying, and stabilizing agent in nutraceutical and pharmaceutical products [5]. Recently, this seaweed was reported to have beneficial effects in preventing diet-induced metabolic syndrome [6]. Other research has shown that* K*.* alvarezii* is cardioprotective [7] and has wound healing [8], antioxidant [9], antimicrobial [10], and anti-inflammatory [11] properties. Pangestuti and Kim [12] reported that* K*.* alvarezii* has antioxidant, anti-neuroinflammatory, and anti-cholinesterase activities. Previously, we showed that* K*.* alvarezii* exerts its neurotrophic activity by accelerating the initial neuronal maturation and stimulating axodendritic arborization in primary cultures of hippocampal neurons [13, 14].

In Indonesia,* K*.* alvarezii* is abundant and is cultivated in many localities. Generally, the bioactive compound content of macroalgae depends on physical and biological factors, such as climate, water quality, reproductive state, blade age, thallus section, locality, and seasonality [15]. The biochemical composition of* K*.* alvarezii* can vary with the cultivation period and planting density [16], depth [17], season [18], and spatiotemporal conditions [19]. It is important to evaluate the growth conditions and bioactivity when macroalgae are exploited commercially. Studies of the depth, growth period, and locality are essential when characterizing the bioactivities of algae.

Therefore, we cultivated* K*.* alvarezii* thalli at different depths and for different growth periods in various areas of Indonesia and compared the neurite outgrowth-promoting activity on hippocampal neuron cells.

## 2. Materials and Methods

### 2.1. Macroalgal Materials and Aquaculture

**Figure 1:**
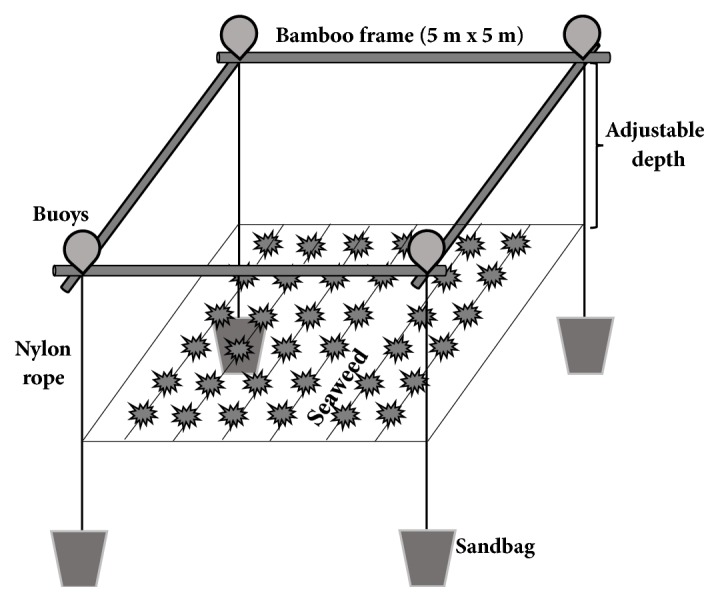
The floating bamboo frame system used in this study. The bamboo frame measured 5 m × 5 m. Six culture nets were fixed to the frame. The depth of the culture nets was adjusted to 0, 1, or 2 m. Six algal thalli were grown on each culture line (5 m).

The floating bamboo method was used for aquaculture, with the cages placed 200 meters from the shore. The bamboo frames (5 × 5 m^2^) were equipped with polyethylene plastic buoys for floatation and sandbags for anchors to prevent the influence of tides (Figure 1). Nylon lines were connected to the bamboo frame vertically and horizontally. The depth of nylon culture nets was adjusted to 0, 1, or 2 meters. At each depth, a culture net was used for macroalgae cultivation. Approximately 200 g (wet weight) of fresh* K*.* alvarezii* thalli was tied to the culture ropes with plastic twine. Tissue samples were collected after 0, 15, 30, or 45 days, rinsed twice with seawater to remove debris, and rinsed again with freshwater to remove salt. Then, the tissues were dried in the shade for 2~3 days. The dried samples were pulverized into a powder in a grinder (HMF-340, Hanil, Seoul, Korea) and kept in the dark at –20°C until used.

Thalli of the carrageenophytes* K*.* alvarezii*,* K*.* striatum*, and* Eucheuma denticulatum* were cultured in Buleleng Regency (8°11′01.5′′ S, 114°48′39.2′′ E), western Bali, Indonesia, during the dry seasons (April–June) from 2015 to 2017. The three carrageenophytes were cultured on nets at the surface (0 m depth) for 45 days. For the experiments at different depths and for growing periods,* K*.* alvarezii* was cultivated at 0, 1, and 2 m for 0, 15, 30, and 45 days at the same site in Buleleng Regency. For the experiments comparing location,* K*.* alvarezii* thalli were cultured at the surface (0 m depth) in cages of the same size for 45 days at 14 localities in Indonesia: Bali, Banten, Batam, Garut, Kalimantan, Karimunjawa, Kendari, Lampung, Lombok, Morotai, Papua, Sumbawa, Takalar, and Ternate.

### 2.2. Culture and Treatment of Primary Hippocampal Neurons

Cultures of hippocampal neurons were prepared in 24-well polystyrene plates. All cell culture reagents were purchased from Invitrogen (Carlsbad, CA, USA) unless otherwise stated. All animal care and use were in agreement with institutional guidelines and approved by the Institutional Animal Care and Use Committee of the College of Medicine, Dongguk University, Korea. Primary hippocampal neurons were prepared as described previously [20, 21]. Briefly, Sprague-Dawley rats on day 19 of pregnancy were euthanized with isoflurane, and the fetuses were collected. The neuronal cells dissociated from the fetal hippocampi were counted with a hemocytometer and plated at a density of approximately 1–2 × 10^4^ cells/cm^2^ onto poly-DL-lysine-coated glass coverslips in 24-well culture plates. Cultures were maintained in serum-free neurobasal media supplemented with B27 and incubated at 37°C under 5% CO_2_ and 95% air for 2 days* in vitro* (DIV 2).

Fine powder of* K. alvarezii* was processed to obtain an extract using 95% ethanol according to Tirtawijaya et al. [13]. The ethanol extract was dissolved in dimethyl sulfoxide (DMSO) to 8 mg/ml. The extract with an optimal concentration of 1 *μ*g/ml or vehicle control (DMSO, final concentration ≤ 0.5%) was added to the culture media prior to cell plating.

### 2.3. Image Acquisition

Images (1,388 × 1,039 pixels) were captured using a Leica DM IRE2 research microscope equipped with I3 S, N2.1S, and Y5 filter systems (Leica Microsystems AG, Wetzlar, Germany) and a high-resolution CoolSNAP™ CCD camera (Photometrics, Munich, Germany) under the control of a computer using the Leica FW4000 program. The digital images were processed using Adobe Photoshop 7.0 software (Adobe, San Jose, CA, USA).

### 2.4. Image Analysis and Quantification

Morphometric analyses and quantification were performed using ImageJ software, version 1.48 (National Institutes of Health, Bethesda, MD, USA; http://imagej.nih.gov/ij) with the simple neurite tracer plug-in. Morphometric parameters such as the number of primary neurites (NPN; neurites that originated directly from the soma), the total length of primary neurites (TLPN; the sum of primary neurite lengths), and the length of the longest neurite (LLN) were measured. Neurons (a minimum of 50 cells) that were not intermingled with the processes of adjacent neurons were selected for analysis. We always compared the extract-treated cultures with the vehicle control (cultures with DMSO).

### 2.5. Statistical Analysis

Results are shown as the means ± standard error (SE) of at least three independent experiments. Data were checked for the normality of the distribution by the Kolmogorov-Smirnov test, and normally distributed data were used for further analysis. We conducted a two-way analysis of variance (ANOVA) on data of different depths and culture times, while data from different species and localities were analyzed by a one-way ANOVA. Duncan's post hoc multiple comparison was used to determine significant (*P *< 0.05) differences among ≥ 3 treatment means. Correlations between cultivation conditions and NPN, TLPN, and LLN were examined using Pearson's correlation coefficients with *α* < 0.05. All calculations were carried out using SPSS statistical software for Windows, version 17.0 (SPSS Inc., Chicago, IL, USA).

## 3. Results

**Figure 2:**
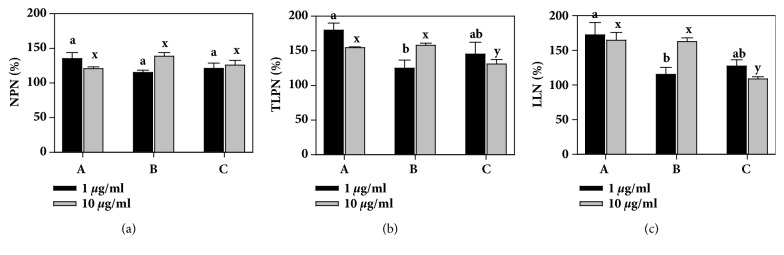
Neurotrophic activity of* Kappaphycus alvarezii* (A),* Kappaphycus striatum* (B), and* Eucheuma denticulatum* (C) on primary hippocampal neurons at day* in vitro* (DIV) 2. The seaweeds were grown at the surface for 45 days in west Bali. The morphometry of the neurons (≥ 50 cells) was measured based on (a) the number of primary neurites (NPN), (b) the total length of the primary neurites (TLPN), and (c) the length of the longest neurite (LLN). Activity (%) is expressed as the mean ± SE (*n* ≥ 3) relative to the control. Extracts of 1 *μ*g/ml (black) and 10 *μ*g/ml (grey) were added to the neuron cultures. Different letters at the 1 *μ*g/ml (a, b) and 10 *μ*g/ml (x, y) concentrations indicate significant differences (*P* < 0.05).

Neurotrophic activity was quantified based on morphometric analyses of hippocampal neurons using NPN, TLPN, and LLN. Adding the* K*.* alvarezii* extract to the neuron cultures enhanced the growth of neurites. First, we compared three abundant carrageenophytes (*K*.* alvarezii*,* K*.* striatum*, and* E*.* denticulatum*) that are aquaculturable in Indonesia to select the one with the highest neurotrophic activity. We grew the plants at the surface (0 m depth) for 45 days on bamboo frame nets in Buleleng Regency and compared their neurotrophic activities (Figure 2). The three species did not differ significantly in terms of NPN, but they differed significantly in terms of TLPN and LLN (*P *< 0.05).* K*.* alvarezii* had higher neurotrophic activity in terms of TLPN and LLN than* K*.* striatum* or* E*.* denticulatum*, so* K*.* alvarezii* was selected as the best species for further neurotrophic experiments.

Next, to determine the effective growth conditions to promote the neurotrophic activity of* K*.* alvarezii*, thalli were grown at different depths and for different periods. The* K*.* alvarezii* grown at 0 m for 45 days and at 1 m for 30 days had a significantly higher NPN, increasing by approximately 1.4- and 1.4-fold, respectively, compared with the vehicle control (Table 1). The initial seeds at 0 d, harvested from thalli grown for 45 days, had an NPN 1.2-fold higher than the control. After growth for 15 days at all depths, i.e., the young growing stage, neurotrophic activity was similar to that of the controls; i.e., there was no neurotrophic activity. NPN tended to be lower in cultures at a depth of 2 m for all harvest times.

In terms of TLPN, the seaweed thalli grown at 0 m for 45 days and at 1 m for 30 days had significantly higher activity than the control, increasing by approximately 1.8- and 1.8-fold, respectively (Table 2). Generally, thalli grown at less than 1 m and for longer than 30 days had high activity. The initial seeds at 0 day, from seaweed previously grown for 45 days, also had a high TLPN. No promotional activity was seen after growth for 15 days at all depths. TLPN tended to be lower for all cultures at a depth of 2 m for all harvest times.

With regard to the promotion of LLN, thalli grown at depth 0 m for 45 days had the highest activity, increasing by approximately 1.7-fold higher than the control (Table 3). Generally, thalli grown at less than 1 m and for longer than 30 days had high activity. The initial seeds at 0 day also had a high LLN. At a culture time of 15 days at all depths, no promotional activities were observed. LLN tended to decrease at a depth of 2 m for all harvest times.

**Figure 3:**
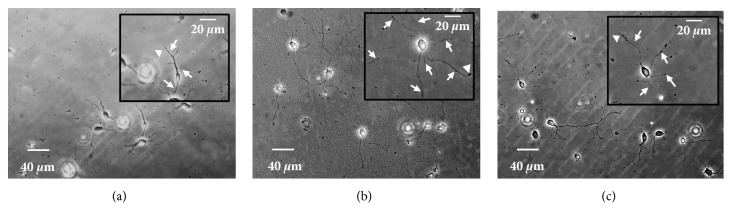
Representative images of neurons grown in vehicle (a) and extracts of* Kappaphycus alvarezii* grown at Ternate (b) and Garut (c). Primary neurites (arrows) and the longest neurite (arrowheads) were observed using ImageJ software to determine the NPN, TLPN, and LLN.

To investigate the influence of location, thalli of* K*.* alvarezii* were cultured at the surface for 45 days in 14 localities in Indonesia. Extracts were prepared from the thalli from the 14 localities and their neurotrophic activities were tested. The NPN, TLPN, and LLN activities in extracts from different areas differed to varying degrees (Table 4). Relatively high activities were detected in thalli cultured at Ternate, North Maluku, and Garut, West Java. Thalli from Ternate promoted NPN, TLPN, and LLN by 1.8-, 2.1-, and 1.9-fold, respectively, compared with the control, while thalli from Garut promoted them by 1.7-, 2.0-, and 2.0-fold. The highest NPN was obtained with the extract from Ternate and the lowest with that from Takalar (1.1-fold the control). The highest TLPN was obtained with the extract from Ternate and the lowest from Batam (1.2-fold). The highest LLN was obtained from Lombok (2.0-fold) and the lowest from Batam (1.1-fold). The neurotrophic activity of the* K*.* alvarezii* extracts varied significantly with location (*P* < 0.05). These results confirmed that different environmental conditions affect the production of neurotrophic compounds in* K*.* alvarezii* tissues.  Figure 3 shows representative neurons grown in vehicle and extracts of* K*.* alvarezii* cultivated in Ternate and Garut on DIV 2.

## 4. Discussion

Neurotrophic factors are molecules that are critical for neurite outgrowth and affect neuron survival, synaptic plasticity, and the formation of long-lasting memories [22]. Previously, we found that the rhodophyte* K*.* alvarezii* was the best candidate for the production of neurotrophic factors from among 34 common Indonesian macroalgae tested [13]. This macroalga contains several lipophilic compounds that contribute to its neurotrophic activity [14]. There is high demand for* K*.* alvarezii* aquaculture in Indonesia for the production of hydrocolloid carrageenan.* K*.* striatum* and* E*.* denticulatum*, two other carrageenan producers, are also widely cultivated in Indonesia. The genus* Kappaphycus* produces kappa-carrageenan, which has greater gel strength and 3,6-anhydrogalactose levels and fewer sulfate radicals than iota-carrageenan [23]. The genus* Eucheuma* produces iota-carrageenan. Comparison of these three common carrageenophytes revealed that* K*.* alvarezii* had greater neurotrophic activity than the others. The high neurotrophic activity of* K*.* alvarezii* did not appear to be related to the gel strength of carrageenan or the amount of sulfate or anhydrogalactose moieties in carrageenan. Some research has indicated that the antioxidant activity and total phenol and carotene contents differ between* E*.* cottonii* (now known as* K*.* alvarezii*) and* E*.* spinosum* (now known as* E*.* denticulatum*) [24], but Rosni et al. [25] reported that several varieties of* K*.* alvarezii*,* K*.* striatum*, and* E*.* denticulatum* had insignificant protein and total phenolic contents. Therefore, the neurotrophic activity is not directly related to the levels of antioxidants, phenolics, carotene, or proteins.

Two common methods are used for carrageenophytes aquaculture: longlines and floating cages. A floating cage results in higher biomass production than a longline because the macroalgae can be cultivated vertically in more space and protected from herbivores [26]. Given that light decreases exponentially as depth increases, the chlorophyll and carotenoid pigments increase with depth [17]. We found that the neurotrophic activity was highest in* K*.* alvarezii* grown at the surface. Therefore, the neurotrophic activity is also not related to pigment production. The culture conditions that yield the best carrageenan from a commercial perspective were 45 days growth at the surface [16]. These culture conditions were optimal for the production of both carrageenan and neurotrophic compounds in* K*.* alvarezii*. When grown for longer than 45 days, thalli are prone to breaking and are lost [16].

The values of NPN, TLPN, and LLN varied from 111.19 to 181.09%, 117.51 to 208.49%, and 105.80 to 199.06%, respectively, at all sites studied. Significant spatial differences were observed in NPN, TLPN, and LLN (one-way ANOVA, all* P* ≤ 0.05), indicating that different environmental conditions affected the neurotrophic activity. The concentrations of secondary metabolites in macroalgae may vary with changes in environmental conditions [15, 27]. Overall, the neurotrophic activity of* K*.* alvarezii* from all localities studied was better than the vehicle control. Therefore, the carrageenophyte* K*.* alvarezii* produces neurotrophic compounds under various environmental conditions, although some conditions are more optimal. The macroalgae studied came from all regions of Indonesia, suggesting that* K*.* alvarezii* can be cultivated in various localities. Future work should examine the relationship between culture conditions in different localities and the composition of neurotrophic compounds in* K*.* alvarezii* tissues.

## 5. Conclusions

Neurotrophic activities from the carrageenophyte* K. alvarezii* (Rhodophyta) were measured in primary cultures of rat hippocampal neurons. This seaweed had higher neurotrophic activity than the other carrageenophytes,* K*.* striatum* and* Eucheuma denticulatum, *cultured under the same conditions.* K*.* alvarezii* grown at the surface for 45 days had higher neurotrophic activity than thalli grown at a depth of 2 m or harvested sooner (15 days). Relatively high activity levels were detected in thalli cultured at Ternate and Garut, Indonesia. Thus, from a commercial perspective, the culture conditions at the surface for 45 days were optimal for the production of both neurotrophic compounds and carrageenan.

## Figures and Tables

**Table 1:** Comparison of the number of primary neurites (NPN) in hippocampal neurons exposed to extracts of *K*. *alvarezii* grown at different depths and for different culture periods.

Depth (m)	Culture period (days)			
	0	15	30	45
0	121 ± 3_ _^abx^	107 ± 4_ _^ax^	126 ± 5_ _^bxy^	135 ± 9_ _^bx^
1	121 ± 3_ _^abx^	105 ± 2_ _^bx^	140 ± 2_ _^cx^	122 ± 8_ _^ax^
2	121 ± 3_ _^abx^	110 ± 3_ _^ax^	122 ± 5_ _^by^	118 ± 3_ _^abx^

Activity (%) is expressed as the mean ± SE (*n* ≥ 3) relative to the control. Day 0: initial seeding day. Means within each column with different letters (x, y) differ significantly (*P* < 0.05). Means within each row with different letters (a–c) differ significantly (*P* < 0.05).

**Table 2:** Comparison of the total length of primary neurites (TLPN) in hippocampal neurons exposed to extracts of *K*. *alvarezii* grown at different depths and for different culture periods.

Depth (m)	Culture period (days)			
	0	15	30	45
0	158 ± 5_ _^ax^	115 ± 6_ _^bx^	160 ± 5_ _^ax^	180 ± 10_ _^ax^
1	158 ± 5_ _^ax^	100 ± 1_ _^bx^	177 ± 6_ _^ax^	164 ± 17_ _^axy^
2	158 ± 5_ _^ax^	100 ± 7_ _^bx^	123 ± 6_ _^cy^	131 ± 2_ _^cy^

Activity (%) is expressed as the mean ± SE (*n* ≥ 3) relative to the control. Day 0: initial seeding day. Means within each column with different letters (x, y) differ significantly (*P* < 0.05). Means within each row with different letters (a–c) differ significantly (*P* < 0.05).

**Table 3:** Comparison of the length of the longest neurites (LLN) in hippocampal neurons exposed to extracts of *K*. *alvarezii* grown at different depths and for different culture periods.

Depth (m)	Culture period (days)			
	0	15	30	45
0	153 ± 7_ _^ax^	105 ± 7_ _^bxy^	154 ± 5_ _^ax^	172 ± 18_ _^ax^
1	153 ± 7_ _^ax^	101 ± 5_ _^bxy^	160 ± 5_ _^ax^	155 ± 12_ _^ax^
2	153 ± 7_ _^ax^	93 ± 5_ _^by^	106 ± 6_ _^bcy^	122 ± 2_ _^cy^

Activity (%) is expressed as the mean ± SE (*n* ≥ 3) relative to the control. Day 0: initial seeding day. Means within each column with different letters (x, y) differ significantly (*P* < 0.05). Means within each row with different letters (a–c) differ significantly (*P* < 0.05).

**Table 4:** Neurotrophic activities of extracts of *K*. *alvarezii* thalli grown in various areas in Indonesia.

Culture area	Activity (%)		
	NPN	TLPN	LLN
West Bali, Bali	135 ± 9_ _^ab^	180 ± 10_ _^a^	172 ± 18_ _^ab^
Banten, Banten	149 ± 4_ _^bc^	200 ± 11_ _^ab^	184 ± 4_ _^ab^
Batam, Riau Islands	135 ± 8_ _^ab^	118 ± 7_ _^c^	106 ± 6_ _^c^
Garut, West Java	169 ± 3_ _^cd^	196 ± 10_ _^ab^	198 ± 12_ _^b^
Bunyu Island, North Kalimantan	164 ± 4_ _^cd^	183 ± 3_ _^ab^	189 ± 10_ _^ab^
Karimunjawa, Central Java	138 ± 2_ _^ab^	144 ± 10_ _^cde^	141 ± 12_ _^de^
Kendari, Southeast Sulawesi	152 ± 12_ _^bc^	186 ± 6_ _^ab^	162 ± 9_ _^ae^
Lampung, Lampung	149 ± 9_ _^bc^	175 ± 10_ _^a^	177 ± 7_ _^ab^
Lombok, West Nusa Tenggara	154 ± 4_ _^bc^	187 ± 4_ _^ab^	199 ± 8_ _^b^
Morotai, North Maluku	171 ± 9_ _^cd^	185 ± 5_ _^e^	152 ± 13_ _^ab^
Papua, Papua	120 ± 1_ _^ae^	132 ± 5_ _^cde^	118 ± 5_ _^cd^
Sumbawa, West Nusa Tenggara	135 ± 4_ _^ab^	148 ± 9_ _^de^	140 ± 6_ _^de^
Takalar, South Sulawesi	111 ± 2_ _^e^	121 ± 2_ _^cd^	136 ± 7_ _^de^
Ternate, North Maluku	181 ± 13_ _^d^	209 ± 11_ _^b^	189 ± 11_ _^ab^

Thalli were cultured at the surface for 45 days in 14 different locations. Activity (%) is expressed as the mean ± SE (*n* ≥ 3) relative to the control. Means within each column with different letters (a–e) differ significantly (*P* < 0.05).

## Data Availability

The data used to support the findings of this study are available from the corresponding author upon request.
